# Potential of IL-1, IL-18 and Inflammasome Inhibition for the Treatment of Inflammatory Skin Diseases

**DOI:** 10.3389/fphar.2017.00278

**Published:** 2017-05-22

**Authors:** Gabriele Fenini, Emmanuel Contassot, Lars E. French

**Affiliations:** Department of Dermatology, University Hospital ZurichZurich, Switzerland

**Keywords:** IL-1, IL-1alpha, IL-1beta, IL-18, inflammatory skin conditions, inflammasome

## Abstract

In 2002, intracellular protein complexes known as the inflammasomes were discovered and were shown to have a crucial role in the sensing of intracellular pathogen- and danger-associated molecular patterns (PAMPs and DAMPs). Activation of the inflammasomes results in the processing and subsequent secretion of the pro-inflammatory cytokines IL-1β and IL-18. Several autoinflammatory disorders such as cryopyrin-associated periodic syndromes and Familial Mediterranean Fever have been associated with mutations of genes encoding inflammasome components. Moreover, the importance of IL-1 has been reported for an increasing number of autoinflammatory skin diseases including but not limited to deficiency of IL-1 receptor antagonist, mevalonate kinase deficiency and PAPA syndrome. Recent findings have revealed that excessive IL-1 release induced by harmful stimuli likely contributes to the pathogenesis of common dermatological diseases such as acne vulgaris or seborrheic dermatitis. A key pathogenic feature of these diseases is IL-1β-induced neutrophil recruitment to the skin. IL-1β blockade may therefore represent a promising therapeutic approach. Several case reports and clinical trials have demonstrated the efficacy of IL-1 inhibition in the treatment of these skin disorders. Next to the recombinant IL-1 receptor antagonist (IL-1Ra) Anakinra and the soluble decoy Rilonacept, the anti-IL-1α monoclonal antibody MABp1 and anti-IL-1β Canakinumab but also Gevokizumab, LY2189102 and P2D7KK, offer valid alternatives to target IL-1. Although less thoroughly investigated, an involvement of IL-18 in the development of cutaneous inflammatory disorders is also suspected. The present review describes the role of IL-1 in diseases with skin involvement and gives an overview of the relevant studies discussing the therapeutic potential of modulating the secretion and activity of IL-1 and IL-18 in such diseases.

## Interleukin-1 Family

Cytokines comprise a variety of molecules secreted by immune and non-immune cells that regulate important cellular functions and physiological processes especially in the hematopoietic and immune systems. One important class of cytokines are the interleukins, a large family of small secreted proteins that bind to specific membrane receptors on target cells. The history of interleukins, and particularly of interleukin-1 (IL-1), began in 1948 when Paul B. Beeson discovered an active unknown substance obtained from rabbit leukocytes that was able to cause fever (Beeson, 1948). Decades later, Dinarello et al. (1974, 1977) identified two chemically and biologically distinct pyrogenic molecules produced by neutrophils and monocytes incubated with heat-killed *Staphylococcus albus*; he named them human leukocytic pyrogens (LP). Before him, Gery et al. (1972) reported that the stimulation of murine and human lymphocytes with lipopolysaccharide (LPS), an essential component of Gram-negative bacteria, led to the release of a soluble factor that was able to enhance the response of T lymphocytes to lectins (phytohemagglutinin and concanavalin A). In 1979, the molecules with inflammatory properties reported by Charles Dinarello and Igal Gery revealed to be the same, namely IL-1 (Rosenwasser et al., 1979). Following progress in sequencing technologies, it turns out that the IL-1 family comprises a total of eleven members with similar or distinct biological effects. In addition to IL-1α and β, IL-18, IL-33, IL-36α, β and γ are pro-inflammatory, whilst, IL-1 receptor agonist (IL-1Ra), IL-36Ra, IL-37, and IL-38 are anti-inflammatory. Genes encoding IL-1 family members are mostly located on human chromosome 2 with two exceptions, namely the genes encoding IL-18 and IL-33 that are located on chromosomes 11 and 9, respectively.

IL-1β is not only secreted by immune cells such as monocytes/macrophages, dendritic cells, neutrophils, B lymphocytes and NK cells but also by non-immune cells such as keratinocytes (Dinarello, 2009; Feldmeyer et al., 2010). IL-1β is able to act on a broad range of cell types (Dinarello, 2009, 2011). It is a key mediator of the acute phase of inflammation inducing local and systemic responses. Its effects are numerous and include the secretion of downstream pro-inflammatory mediators such as cyclooxygenase type-2 (COX-2), IL-6, Tumor Necrosis Factor (TNF) and IL-1 itself (Dinarello, 1996; Weber et al., 2010). In the body, the inflammatory effects of IL-1 manifest as fever, vasodilation and hypotension as well as an increased sensitivity to pain. The pyrogenic activity of IL-1 is due to the activation of NF-κB and the resulting expression of COX-2, an enzyme involved in the synthesis of prostaglandins (Lee et al., 2004).

IL-1 cytokines bind to and act through specific receptors, which are characterized by intracellular Toll/Interleukin-1 receptor (TIR) domains and an extracellular immunoglobulin-like binding domain (Boraschi and Tagliabue, 2013). The IL-1 receptor family comprises several members including IL-1R1, the decoy receptor IL-1R2, IL-1R accessory protein (IL-1RaP or IL-1R3), IL-1R4 (T1 or ST2), IL-18Rα (IL-1R5), IL-36R (IL-1R6), IL-18R accessory protein (IL-18Rβ or IL-1R7), IL-1R8 (TIR8), IL-1R9 (IL-1RAPL2), and IL-1R10 (TIGIRR).

IL-1α/IL-1β, IL-18, and IL-36 initiate immune and inflammatory responses by binding to IL-1R1, IL-18Rα, and IL-36R, respectively. The co-receptor IL-1RaP interacts with IL-1R1, IL-1R2, IL-1R4, and IL-36R while IL-18Rβ is a unique accessory chain for IL-18Rα. The decoy receptor IL-1R2 lacks the cytoplasmic TIR domains and is therefore unable to initiate a signaling cascade even in the presence of its accessory receptor. IL-1R2 binds IL-1β with high affinity and IL-1α or IL-1Ra with low affinity (Symons et al., 1995). The biological activity of IL-1 family cytokines is tightly regulated not only by decoy receptors but also by soluble receptor antagonists such as IL-1Ra and IL-36Ra that can specifically antagonize IL-1α, IL-1β, and IL-36. In addition, IL-1R1 can be released into the extracellular space where, in its soluble form (sIL-1R1), it can also function as a soluble decoy receptor and prevent the binding of IL-1α, IL-1β, and IL-1Ra to membrane IL-1R1 (Burger et al., 1995). Furthermore, IL-1R2 can also be cleaved and solubilized by metalloproteinases resulting in an increased segregation of IL-1β due to its higher affinity (Symons et al., 1995).

First identified and described as interferon-γ-inducing factor (IGIF) (Nakamura et al., 1989, 1993), IL-18 received its current name 3 years later (Ushio et al., 1996). In contrast to the strong pyrogenic activity of IL-1α and IL-1β, IL-18 is only able to induce fever at higher concentrations. IL-18 activates primarily p38 MAPK and AP-1, but fails to activate NF-κB (Lee et al., 2004). IL-18 activity is mainly regulated by a soluble protein called IL-18 binding protein (IL-18BP). IL-18BP differs from the other soluble IL-1 receptors because it retains a unique binding sequence composed by a single immunoglobulin domain (Dinarello et al., 2013). Similar to the IL-1Rs, IL-18Rs can also be found in a soluble form that is used as a biomarker for inflammatory diseases such as rheumatoid arthritis (RA) and adult-onset Still’s disease (AoSD) (Takei et al., 2011).

Both IL-1β and IL-18 are first synthesized as precursors which need to be processed into their biologically active form by a cytoplasmic protein complex known as the inflammasome. In contrast, both pro and cleaved forms of IL-1α are biologically active and induce, via IL-1R1 signaling, the production of TNFα and IL-6 in human A549 epithelial cells and peripheral blood mononuclear cells (PBMCs) (Kim et al., 2013). The transcription of the IL-1α gene is regulated by a variety of stimuli including proinflammatory or stress-associated stimuli and growth factors (Di Paolo and Shayakhmetov, 2016). Pro-IL-1α lacks a signal secretion peptide, however, its release from dying cells is able to trigger acute inflammation (Chen et al., 2007). IL-1α can be translocated to the plasma membrane where it signals in an intra and paracrine manner but can also be secreted in its mature form via both IL-1β-dependent or independent pathways (Fettelschoss et al., 2011; Gross et al., 2012). Since pro-IL-1α contains a nuclear localization signal, it can induce the expression of proinflammatory genes independently of IL-1R1 signaling (Werman et al., 2004). Because of the multiplicity of mechanisms of action of IL-1α, it plays an important role in the maintenance of homeostasis and the pathology of several human diseases (Di Paolo and Shayakhmetov, 2016).

## Pathogen Recognition Receptors

Pathogen recognition is fulfilled by a distinct set of receptors, the so-called pathogen-recognition receptors (PRRs). These include C-type lectin receptors (CLRs), Toll-like receptors (TLRs), retinoic acid-inducible gene I (RIG-I)-like receptors (RLRs), nucleotide-binding oligomerization domain (NOD)-like receptors (NLRs), AIM2-like receptors (ALRs), and partially the complement system. Such PRRs have different localizations and ligands. For example: the complement system acts as an extracellular sensor for conserved pathogen motives (carbohydrates) and host antibodies; TLRs are found on cellular membrane and endosomes and bind to a variety of molecules including nucleic acids (TLR3, TLR8, TLR9, and TLR13), small proteins (TLR2, TLR4, TLR11, and TLR12), lipopeptides and lipoproteins (TLR1, TLR2, and TLR6), glycolipids (TRL2, TLR4) and small drugs (TLR4, TLR7) (Leifer and Medvedev, 2016). CLRs are either localized at the cell surface or in endosomes and primarily bind to carbohydrates (mannose, fucose, GlcNAc, and β-1,3-glucan) in a Ca^2+^-dependent manner but the recognition of proteins, lipids and inorganic compounds like CaCO_3_ has also been reported (Zelensky and Gready, 2005).

Retinoic acid-inducible gene I (RIG-I)-like receptors and NLRs are exclusively located in the cytosol. RLRs include RIG-I, MDA5 and the co-receptor LGP2. They are specialized in the sensing of viral double-stranded RNA (Reikine et al., 2014). NLRs constitute an expanding family of receptors able to detect a variety of molecules. They are composed of several domains: the central NOD or nucleotide-binding domain (NBD) that includes a NTPase NACHT domain controlling self-oligomerization, and the leucine-rich repeat (LRR) domain involved in ligand sensing (Schroder and Tschopp, 2010). On their N-terminal extremity, NLRs have either a pyrin domain (PYD), a caspase-recruitment domain (CARD) or a baculoviral inhibition of apoptosis protein repeat domain (BIR) and are consequently named NLRPs, NRLCs or NAIPs, respectively.

## The Inflammasomes

In 2002, the group of Prof. Tschopp described a multiprotein complex able to oligomerize and activate inflammatory caspases leading to the processing of IL-1β and IL-18 (Martinon et al., 2002). This complex was named **NLR PYD-containing protein 1 (NLRP1)-inflammasome** and was shown to contain the scaffold NLRP1 interacting via PYD with the adaptor protein apoptosis-associated speck-like protein containing a CARD (ASC, also known as PYCARD) which can then recruit the inflammatory procaspase, caspase-1(also known as IL-1β-converting enzyme or ICE). Upon sensing of appropriate ligands and subsequent inflammasome activation, procaspase-1 is autocatalytically cleaved and activated (Wilson et al., 1994). Active caspase-1 can then process IL-1β and IL-18 and the biologically active cytokines are secreted in an unconventional golgi/endoplasmic reticulum-independent manner (Keller et al., 2008). The NLRP1 inflammasome is able to sense bacterial peptidoglycan muramyl dipeptide (MDP) (Hsu et al., 2008) and can be activated in keratinocytes by ultraviolet B (UVB) irradiation (Feldmeyer et al., 2007). Mutations in the *Nlrp1* gene have been linked to susceptibility to vitiligo-associated autoimmune diseases (Jin et al., 2007), systemic lupus erythematosus (SLE) and RA (Masters, 2013). Gain-of-function mutations of the *NLRP1* gene were recently described in two skin disorders, namely multiple self-healing palmoplantar carcinoma (MSPC) and familial keratosis lichenoides chronica (FKLC). *NLRP1* mutations result in the blockade of the autoinhibitory effect of NLRP1 PYD domain and lead to an increased activation of the inflammasome (Zhong et al., 2016). NLRP1 also contains a C-terminal CARD domain which mediates direct interaction with caspase-1. A recent study has demonstrated that anthrax lethal factor can cleave the PYD domain of murine but not human NLRP1 causing its activation. This identifies proteolysis as an alternative activation mechanism for NLRP1 (Chavarria-Smith et al., 2016).

The **NLRP3 inflammasome** is the best characterized inflammasome to date, and a broad range of stimuli can induce its activation. These include PAMPs such as LPS, fungal zymosan, bacterial toxins, and also the bacteria *Listeria monocytogenes* (Meixenberger et al., 2010), *S. aureus* (Munoz-Planillo et al., 2009), and *Propionibacterium acnes* (Kistowska et al., 2014b; Qin et al., 2014), as well as yeasts like *Candida albicans* (Hise et al., 2009) and of the *Malassezia* spp. (Kistowska et al., 2014a). NLRP3 can also be activated by danger-associated molecules that are not derived from pathogens but often associated with cellular stress, the so-called DAMPs, including extracellular ATP (Mariathasan et al., 2006), asbestos (Dostert et al., 2008), amyloid-β (Halle et al., 2008), DNA:RNA hybrids (Kailasan Vanaja et al., 2014), and crystals such as gout-causing monosodium urate (MSU) (Martinon et al., 2006), silica (Dostert et al., 2008), or cholesterol (Duewell et al., 2010). Interestingly, the study of patients with autosomal dominant cold-induced urticaria, later termed familial cold autoinflammatory syndrome (FCAS), allowed the identification of mutations in the *CIAS1/*cryopyrin*/NLRP3* gene (Hoffman et al., 2001). These studies permitted major advances in the identification and understanding of autoinflammatory diseases but also resulted in a gain of interest in IL-1β biology and its role in inflammatory disorders.

**FIGURE 1:**
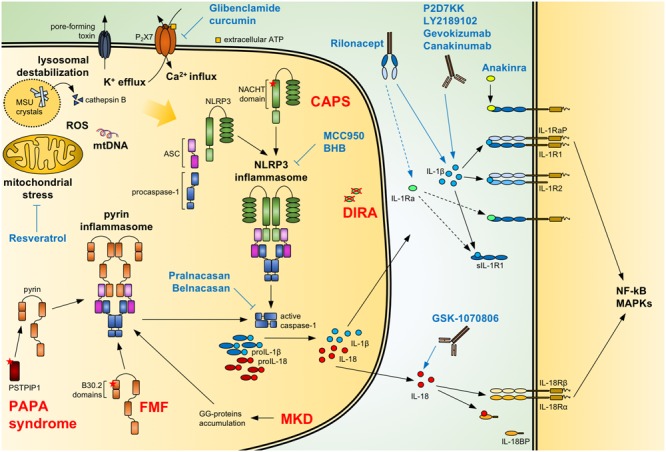
**Regulation of IL-1/18 production and current IL-1/18 antagonists.** Pathogen- and danger-associated molecular patterns can induce the formation of a functional inflammasome via events including mitochondrial stress with release of oxidized mtDNA, ROS, lysosomal destabilization with cathepsin B release, changes in intracellular calcium (Ca^2+^) and potassium (K^+^) efflux. Also, mutations of NACHT-PYD domains of NLRP3 in cryopyrin-associated periodic syndromes (**CAPS**) lead to the activation of the NLRP3 inflammasome. Similarly, mutations of B30.2 domains in familial Mediterranean fever (**FMF**) and of PSTPIP1 in **PAPA syndrome** or accumulation of geranylgeranylated proteins (GG-proteins) in mevalonate kinase deficiency (**MKD**) lead to activation of the pyrin inflammasome. Activated inflammasomes recruit procaspase-1 via the adaptor ASC. Autocatalytically cleaved and activated caspase-1 can then process IL-1β and IL-18. The secreted cytokines bind to the IL-1 receptor (IL-1R1:IL-1RaP) and IL-18 receptor (IL-18Rα:IL-18Rβ), respectively, resulting in NF-κB and MAPKs signaling. Soluble IL-1R1 (sIL-1R1), IL-1R2 and IL-18 binding protein (IL-18BP) can block the signaling pathway. Caspase-1 activity can be modulated by the specific inhibitors **Pralnacasan** and **Belnacasan**. The activation of the NLRP3 inflammasome can be inhibited with **Glibenclamide** and **curcumin** inhibiting or downregulating the ATP-sensitive K^+^-channels P_2_X7, respectively; with **Resveratrol** increasing autophagy and inhibiting mitochondrial stress; and with the specific NLRP3 inhibitors **MCC950** and **BHB**. **Anakinra** and the secreted IL-1 receptor antagonist (IL-1Ra), absent in deficiency of IL-1 receptor antagonist (**DIRA**) patients, compete with IL-1β and IL-1α (not shown) for binding to the IL-1 receptor. **Rilonacept** acts as a soluble decoy binding IL-1β and with lower affinity IL-1α (not shown) and IL-1Ra. The recombinant antibodies **Canakinumab, Gevokizumab, LY2189102**, and **P2D7KK** specifically target and neutralize IL-1β. Similarly, **GSK-1070806** and **MABp1** inhibits IL-18 and IL-1α (not shown), respectively. Blue arrows: inhibitory effect; dotted arrow: low affinity; ASC, apoptosis associated speck-like domain protein containing a CARD; MSU, monosodium urate; mtDNA, mitochondrial DNA; ROS, reactive oxygen species.

Since such a broad range of stimuli can activate the NLRP3 inflammasome, it is believed that a common mechanism triggered by diverse activators leads to NLRP3 activation. Several events such as the release of oxidized mitochondrial DNA (Shimada et al., 2012), production of reactive oxygen species (ROS) (Dostert et al., 2008), mitochondrial stress (Zhou et al., 2011), lysosomal rupture with cathepsin B release (Hornung et al., 2008), changes in intracellular calcium (Ca^2+^) levels (Murakami et al., 2012) and potassium (K^+^)-efflux (Petrilli et al., 2007) have been reported to be associated to inflammasome activation (**Figure 1**). Whether all or only a part of these events are required for NLRP3 inflammasome activation is not clear. Munoz-Planillo et al. (2013) suggested that the sole reduction of intracellular K^+^ was sufficient for NLRP3 inflammasome activation but recent reports have suggested that, in certain circumstances, inflammasome activation can occur independently of K^+^-efflux (Gross et al., 2016) or phagocytosis of bacteria (Chen et al., 2016). Moreover, the activity of the NRLP3 inflammasome has also been reported to be controlled by kinases such as Bruton’s tyrosine kinase (BTK) interacting with NLRP3 and ASC thus favoring the recruitment of caspase-1 (Ito et al., 2015), and JNK or Syk kinases regulating ASC oligomerization (Hara et al., 2013; Okada et al., 2014). ROS were shown to activate NEK7, a kinase involved in the control of mitosis, causing its direct binding to the LRR domain of NLRP3 and modulating its function (He et al., 2016; Shi et al., 2016). The consensual and unifying mechanism leading to NLRP3 inflammasome is currently a matter of intense debate and investigation.

The **absent in melanoma 2 (AIM2) inflammasome** recognizes viral and bacterial double-stranded DNA (dsDNA) via its PYHIN domain (Muruve et al., 2008). AIM2, like NLRP3, recruits caspase-1 via the adaptor protein ASC. Increased levels of AIM2 were found in keratinocytes of patients with psoriasis and atopic dermatitis, causing acute and chronic skin barrier disruption-related inflammation (Ito et al., 2015).

The **NLRC4 inflammasome** is activated by bacterial flagellin (Mariathasan et al., 2004) and type 3 secretion system proteins (Miao et al., 2010). NLRC4 contains a CARD domain and is therefore able to recruit and activate caspase-1 without the adaptor ASC. *Salmonella typhimurium* is reported to activate NLRC4 by inducing its phosphorylation by protein kinase C δ-type (PKCδ) (Qu et al., 2012). Moreover, NLRC4 can recruit NLRP3 resulting in increased caspase-1 processing (Qu et al., 2016). Reported *NLRC4* gene mutations cause recurrent fever flares and macrophage activation syndrome (MAS) (Canna et al., 2014), neonatal-onset enterocolitis and fatal or near-fatal episodes of autoinflammation (Romberg et al., 2014). A missense mutation of NLRC4 was also associated with FCAS (Kitamura et al., 2014).

The **pyrin inflammasome** is encoded by the *MEFV* gene and contains PYD, TRIM, and B30.2 domains. This inflammasome is activated by bacterial toxins like *Clostridium difficile* toxin B (TcdB) and C3 toxins (Park et al., 2016). Mutations in this gene are the cause of familial Mediterranean fever (FMF) (Chae et al., 2006) and the recently described disease-entity pyrin-associated autoinflammation with neutrophilic dermatosis (PAAND) (Masters et al., 2016). Mevalonate kinase deficiency (MKD) was also linked to the activation of the pyrin inflammasome (Park et al., 2016).

Other less characterized inflammasomes include NLRP2, NLRC2, NLRP6, NLRP7, and NLRP12. Besides their role in caspase-1 activation, they possess roles in activating/inhibiting NF-κB as well as MAPK pathways and autophagy (Lupfer and Kanneganti, 2013).

The involvement of caspase-5 in the processing of IL-1 was already described in the original publication describing the inflammasome (Martinon et al., 2002), but it is only a decade later that its role in cell death was reported (Kayagaki et al., 2011, 2015). Human caspase-4, caspase-5 and the orthologous murine caspase-11 are activated by cytosolic LPS (Shi et al., 2014) and can cleave the substrate gasdermin D (GSDMD). GSDMD creates pores in the cell membrane resulting in pyroptosis, an inflammatory form of programmed cell death (He et al., 2015; Shi et al., 2015). Activated GSDMD can also induce the formation of the NLRP3 inflammasome and subsequent IL-1β and IL-18 secretion. This process known as the **non-canonical inflammasome** activation, can occur either in a caspase-1/inflammasome-dependent or independent manner (Man and Kanneganti, 2016). Indeed, caspase-8, an initiator caspase mainly involved in apoptosis, can be involved in the activation of the NF-κB pathway and IL-1β/IL-18 processing. Recognition by dectin-1 of extracellular fungi such as *Candida albicans* results in the formation of a complex with CARD9, Bcl-10, MALT1, ASC and caspase-8 which, once activated, can directly process IL-1β. Interestingly, dectin-1 dependent internalization of fungi drives instead the NLRP3 inflammasome (Gringhuis et al., 2012). Moreover, activation of caspase-8 through the Fas-signaling pathway can also lead to the direct processing of IL-1β and IL-18 independently of caspase-1 and ASC (Bossaller et al., 2012).

Other enzymes that are described to process IL-1 family members include neutrophil-derived elastase, cathepsin G and proteinase 3 (myeloblastin), mast cell-derived chymase and granzyme B from cytotoxic lymphocytes and natural killer cells (Afonina et al., 2015).

## Inhibition of Il-1 Signaling

### IL-1β Antagonists

Given the key role of IL-1β in inflammatory and autoinflammatory disorders, several IL-1 inhibitors have been developed and evaluated especially in life-threatening autoinflammatory syndromes. To date, the most efficient way to block IL-1 signaling consists of biologics that specifically target IL-1 or IL-1R1 (**Figure 1** and **Table 1**).

**Table 1:** Biologics and inhibitors of IL-1, IL-18, and inflammasome activation.

Name	Trade name	Company	Class	Target	*h*_1/2_	Status
Anakinra	Kineret^®^	Sobi, Inc.	recIL-Ra	IL-1α, IL-1β	4–6 hours	Marketed
Rilonacept	Arcalyst^®^	Regeneron	srR (IL-1Trap)	IL-1α, IL-1β, IL-Ra	∼7.5 days	Marketed
Canakinumab (ACZ855)	Ilaris^®^	Novartis	mAb (IgG1/κ)	IL-1β	23–26 days	Marketed
Gevokizumab (XOMA 052)		XOMA	mAb (IgG2/κ)	IL-1β	22 days	Phase 3^†^ discontinued
LY2189102		Eli Lilly and Co	mAb (IgG4)	IL-1β	16.8 days	Phase 2
P2D7KK		A^∗^STAR	mAb (IgG1)	IL-1β	∼2 weeks^∗^	Preclinical
Pralnacasan (VX-740)		Vertex	SMI	Caspase-1	nd	Phase 2^†^
Belnacasan (VX-765, HMR3480)		Vertex	SMI	Caspase-1	nd	Phase 2^†^
MCC950			SMI	NLRP3	nd	Preclinical
BHB			SMI	NLRP3	nd	Preclinical
Glibenclamide (glyburide)	Generic		SMI	*K*_ATP_	10 hours	Marketed
MABp1	Xilonix^TM^	XBiotech	mAb (IgG1/κ)	IL-1α	8 days	Phase 3^‡^ Phase 2
GSK-1070806		GlaxoSmith-Kline	mAb (IgG1/κ)	IL-18	23–30 days	Phase 2

*srR, soluble recombinant receptor; SMI, small molecule inhibitor; *K*_*ATP*_, ATP-sensitive potassium channels; h_*1/2*_, half-life; nd, not determined. ^∗^Estimation. ^†^Clinical trial was terminated. ^‡^Only for the treatment of colorectal and non-small cell lung cancers.*

**Anakinra** (Kineret^®^; Sobi, Inc.) is a recombinant non-glycosylated homolog of IL-1Ra that competes with both IL-1α and IL-1β for the binding to IL-1R1 thus impairing the recruitment of IL-1RaP and downstream NF-κB/MAPKs signaling. It is the first biologic developed to specifically target IL-1. Anakinra was first approved in 2001 for the treatment of RA. A decade later, its use was extended to the treatment of cryopyrin-associated periodic syndromes (CAPS) in Europe and for the severest form of CAPS, namely chronic infantile neurological cutaneous and articular syndrome (CINCA) in the USA. Anakinra has a short half-life of 4–6 h and therefore common posology requirements are daily subcutaneous injections of 100 mg/day for RA and 1–2 mg/kg/day for CINCA.

**Rilonacept** (Arcalyst^®^; Regeneron) is a long-acting dimeric fusion protein consisting of portions of IL-1R1 and IL-1RaP linked to the Fc portion of human immunoglobulin G1 (IgG1). Rilonacept acts a soluble decoy binding IL-1β, but also IL-1α and IL-Ra, therefore inhibiting their association with cell surface receptors (IL-1Trap). Rilonacept binds three times stronger to IL-1β than to IL-1α and 12 times stronger to IL-1β than to IL-1Ra. It was approved by the FDA in 2008 for the treatment of CAPS including FCAS and Muckle-Wells syndrome (MWS). Rilonacept has a half-life of 6.3–8.6 days which allows a weekly subcutaneous administration of 320 mg (loading dose) followed by weekly injections of half the loading dose.

**Canakinumab** (ACZ885, Ilaris^®^; Novartis) is a human anti-IL1β monoclonal IgG1/κ isotype antibody with a terminal half-life of 23–26 days and can be therefore administered as a single subcutaneous injection every 2 months.

Canakinumab was approved by the FDA in 2009 for the treatment of CAPS and active systemic juvenile idiopathic arthritis. Recently, it received approval from the FDA as first-line treatment for TNF receptor associated periodic syndrome (TRAPS), MKD and FMF (FDA, 2016).

**Gevokizumab** (XOMA 052; XOMA) is a recombinant humanized anti-IL-1β antibody. In contrast to Canakinumab, which neutralizes IL-1β by competing for binding to IL-1R, Gevokizumab modulates IL-1β bioactivity by reducing its affinity for IL-1R1:IL-1RAcP signaling complex (Blech et al., 2013).

The clinical development of this antibody was interrupted in 2016 after a phase 3 clinical trial evaluating Gevokizumab for the treatment of uveitis in patients with Behçet’s disease did not meet the primary endpoint criteria (Xoma, 2015, 2016).

Gevokizumab showed promising results in phase 2 trial on acne vulgaris (Xoma, 2013) but failed to show benefits in the treatment of pyoderma gangrenosum (Xoma, 2016).

**LY2189102** (Eli Lilly and Co) is a high affinity anti-IL-1β humanized monoclonal immunoglobulin G4 with a terminal half-life of 16.8 days (Bihorel et al., 2014). A weekly treatment of patients suffering from type 2 diabetes mellitus (T2DM) with LY2189102 for 3 months resulted in modest reductions in glycated hemoglobin and blood glucose (Sloan-Lancaster et al., 2013). No further studies have been conducted to date.

**P2D7KK** is another neutralizing monoclonal antibody against IL-1β developed by A^∗^STAR researchers in Singapore. It shares the same mechanism of action as Canakinumab but with an *in vitro* neutralization potency that is 11 times higher. P2D7KK has not been evaluated in human subjects yet but has shown promising effects in three different inflammatory animal models (Goh et al., 2014).

Virus-like particles (VLPs)-based vaccination constitutes a novel approach to target cytokines (Assier et al., 2017). Recombinant mutated IL-1β chemically cross-linked to bacteriophage Qβ VLPs (**hIL1βQβ**) was investigated in a phase 1 clinical trial for T2DM resulting in safe production of specific IL-1β antibodies in the treated patients (Cavelti-Weder et al., 2016).

### Inflammasome Inhibitors

Other possibilities to block IL-1β include the targeting of caspase-1 and NLRP3. Two caspase-1 inhibitors have been developed, namely **Pralnacasan** (VX-740) and **Belnacasan** (VX-765, also HMR3480) (Vertex Pharmaceuticals). These orally absorbed compounds are synthetized as prodrugs which are then converted into the active principle, VRT-018858 and VRT-043198, respectively.

Pralnacasan has been evaluated in clinical trials for the treatment of RA and osteoarthritis but due to safety issues its development has been interrupted (Braddock and Quinn, 2004; Vertex, 2007).

Belnacasan was shown to inhibit IL-1β and IL-18 release from PBMCs of FCAS patients *in vitro* (Stack et al., 2005). It induces anti-inflammatory effects in a mouse model of delayed-type hypersensitivity (DTH) (Wannamaker et al., 2007) and it has been evaluated in phase 1 and 2a clinical trials in the setting of epilepsy and psoriasis (Vertex, 2011).

NLRP3 inhibitors include MCC950, β-hydroxybutyrate and glibenclamide. **MCC950** is a small-molecule able to block canonical and non-canonical NLRP3-induced ASC oligomerization without interfering with NLRC4 and AIM2 activity or TLR signaling (Coll et al., 2015). MCC950 has been shown to be effective for the treatment of CAPS in mice harboring activating *Nlrp3* mutations (Coll et al., 2015) and in mouse models of dermal and airway inflammation (Primiano et al., 2016). **β-hydroxybutyrate** (BHB) is an anti-inflammatory molecule that specifically targets NLRP3 activity. In murine models of FCAS and MWS, BHB inhibited constitutive NLRP3 inflammasome activation (Youm et al., 2015). **Glibenclamide** (glyburide), is an anti-diabetic drug used in the treatment of T2DM. It inhibits the ATP-sensitive K^+^ channel and was shown to block the NLRP3 inflammasome activation induced by LPS, ATP, nigericin and silica (Lamkanfi et al., 2009). These inhibitors present a potential advantage in the treatment of CAPS since they specifically target the NLRP3 inflammasome impacting both IL-1β and IL-18 secretion.

**Resveratrol** and **curcumin** are natural polyphenols found in several plants and are able to block IL-1β secretion. Resveratrol was described to inhibit NLRP1, NLRP3, and NLRC4 activation by preventing mitochondrial damage and augmenting autophagy (Chang et al., 2015). Moreover, Resveratrol was shown to directly bind and block COX-2 activity (Zykova et al., 2008) which is known to be involved in NLRP3 activation (Hua et al., 2015). Curcumin has been shown to impair IL-1β secretion in PMA-treated macrophages by downregulating P2X_7_ receptor and thus inhibiting the TLR4/MyD88/NF-κB pathway (Kong et al., 2016).

### IL-1α and IL-18 Blockers

In contrast to IL-1β, the development of IL-1α and IL-18 inhibitors is less advanced. The monoclonal antibody against IL-1α, **MABp1** (Xilonix^TM^; XBiotech) is the only biologic that specifically target this cytokine and it is currently under investigation for the treatment of advanced cancer (Hong et al., 2014; Hickish et al., 2017).

MABp1 has been evaluated for the treatment of psoriasis (Coleman et al., 2015), acne vulgaris (Carrasco et al., 2015), T2DM (Timper et al., 2015) and is currently being evaluated in patients with hidradenitis suppurativa (HS) who were refractory to anti-TNFα treatment (discussed below).

The only agent targeting the cytokine IL-18 is the **GSK-1070806** antibody (GlaxoSmithKline). In a study for the treatment of T2DM, GSK-1070806 did not reveal any improvement in glucose control (McKie et al., 2016). However, neutralization of IL-18 was shown to reduce the severity of dextran sulfate sodium-induced colitis in mice (Siegmund et al., 2001; Sivakumar et al., 2002).

## Skin Diseases With Il-1 Involvement

Keratinocytes are the most abundant cells in the skin and act as a barrier against water loss and entry of pathogens and irritants. Human keratinocytes constitutively express IL-1α, IL-β, and IL-18 and possess all inflammasome components (Feldmeyer et al., 2007).

Inflammation in the skin with extensive release of IL-1β is often associated with neutrophilic infiltration as first line of defense. In the absence of infection, neutrophils can become detrimental for the host by causing tissue damage (Navarini et al., 2016).

### Monogenic Autoinflammatory Diseases

Monogenic autoinflammatory diseases are a rare group of hereditary syndromes with early manifestation in childhood. They present as inflammatory recurrent flares of fever and skin lesions. Neutrophilic dermatosis is the most common pathological hallmark of these syndromes (Lipsker et al., 2016).

**Cryopyrin-associated periodic syndromes** are disorders caused by mutations in the *NLRP3* gene, previously known as cold-induced autoinflammatory syndrome 1, which results in uncontrolled processing of IL-1β and IL-18 (**Figure 1**). CAPS is a spectrum of three syndromes of increasing severity: **familial cold autoinflammatory syndrome** (FCAS, OMIM #120100), **Muckle-Wells syndrome** (MWS, OMIM #191900) and **chronic infantile neurological cutaneous and articular syndrome** (CINCA, OMIM #697115) also known as neonatal-onset multisystem inflammatory disease (NOMID). They phenotypically share episodes of recurring fever, urticaria-like skin-lesions, conjunctivitis and inflammatory joint pain. In MWS and CINCA, progressive hearing loss and eye inflammation occur; in CINCA, the most severe form of CAPS, central nervous system inflammation is the most devastating symptom leading to increased intracranial pressure and aseptic meningitis (Goldbach-Mansky, 2011). The mutations in the *NLRP3* gene causing FCAS, MWS and CINCA were identified long before the discovery of the inflammasome (Hoffman et al., 2001; Aksentijevich et al., 2002). To date, 182 mutations in the *NLRP3* gene have been reported in the online *registry of hereditary autoinflammatory disorders mutations* (Infevers, 2017).

Mouse models for FCAS and MWS were generated by knocking-in NLRP3 with a L351P and A350V mutation, respectively. Mating of these mice with *Il1r1*^-/-^ mice confirmed the pivotal role of IL-1β in the pathogenesis of these diseases but did not completely rescue the phenotype suggesting a possible IL-18 involvement (Brydges et al., 2009). Generation of FCAS mice lacking both IL-1 and IL-18 receptors did not prevent the mice from succumbing to the disease; this could be explained by residual inflammation due to increased pyroptosis (Brydges et al., 2013).

Although mutations in *NLRP3* gene are the major cause of CAPS, mutation in NLRC4 and NLRP12 have also been reported in few cases (Jeru et al., 2008; Kitamura et al., 2014).

Biologics against IL-1β have revealed successful in the treatment of CAPS. In 2003, a remarkable response after 6 months treatment with 100 mg/day of Anakinra, as was reported in two MWS patients (Hawkins et al., 2003). Anakinra was later revealed to be successful for the treatment of FCAS (Hoffman et al., 2004) and CINCA (Lovell et al., 2005). In a first clinical trial, 18 patients with CINCA received injections of 1-2 mg/kg/day Anakinra resulting in a rapid response in all patients (ClinicalTrials.gov Identifier: NCT00069329) (Goldbach-Mansky et al., 2006). Withdrawal of treatment resulted in relapse of the disease within days and re-administration recovered the drug’s effects. Another open-label study proved the efficacy of Anakinra treatment in 5 FCAS patients over a period of 16 months (ClinicalTrials.gov Identifier: NCT00214851) (Ross et al., 2008).

In an open-label study, five patients with FCAS received a 300 mg loading dose of Rilonacept, resulting in improvement of all symptoms within days of drug administration (ClinicalTrials.gov Identifier: NCT00094900) (Goldbach-Mansky et al., 2008). Under treatment with 100 mg/week (max. 320 mg/week) symptoms were under control in all patients for 24 months. In a randomized double-blind, placebo controlled clinical trial, 47 patients with FCAS and MWS were enrolled and injected weekly with 160 mg Rilonacept for 6 weeks. Ninety-six percent of the patients receiving Rilonacept experienced at least a 30% reduction in the mean key symptom score in contrast to 29% of patients receiving placebo (ClinicalTrials.gov Identifier: NCT00288704) (Hoffman et al., 2008).

Neutralization of IL-1β with Canakinumab for the treatment of CAPS was first described in a phase 3 study involving 35 patients. In the first open-label part, all patients received a single subcutaneous 150 mg Canakinumab injection: 34 individuals had a complete response at day 29. In the second, double-blind, placebo-controlled, randomized withdrawal part of the study, all patients receiving the drug remained in remission whereas 13 out of 16 patients receiving placebo experienced a disease flare. In the third and final open-label part, all enrolled patients received Canakinumab and 97% sustained clinical and biochemical remission at the end of the study (ClinicalTrials.gov Identifier: NCT00465985) (Lachmann et al., 2009). Several other studies support the efficacy of Canakinumab in the treatment of CAPS. In an open-label, phase 3 study with 166 patients, 78% of Canakinumab-naïve patients had a complete response and 90% of the assessed patients were relapse-free over the study period (ClinicalTrials.gov Identifier: NCT00685373) (Kuemmerle-Deschner et al., 2011a). In another study, pediatric MWS and CINCA patients achieved complete response within 1 week after the first Canakinumab injection (ClinicalTrials.gov Identifier: NCT00487708) (Kuemmerle-Deschner et al., 2011b). An additional report revealed that treatment with Canakinumab of CINCA resulted in clinical improvement in five out of six patients but none experienced a full remission (ClinicalTrials.gov Identifier: NCT00770601) (Sibley et al., 2015). Recently, a long-term (26 months) open-label study of 19 CAPS patients, revealed that 95% of the patients were relapse-free at the end of the study and the treatment was well tolerated (ClinicalTrail.gov Identifier: NCT00991146) (Yokota et al., 2016). Finally, results from the β*-confident register* enrolling 288 CAPS patients showed sustained safety of Canakinumab over a follow up period of up to 5 years. Eighty-six patients experienced severe adverse reactions but only five discontinued the treatment (Hoffman et al., 2016, meeting abstract) (ClinicalTrail.gov Identifier: NCT01213641).

Extensive studies on the pathogenesis of CAPS have revealed how mutant NLRP3 has defective interaction with the IL-1β maturation inhibitor cyclic AMP (Lee et al., 2012) or with its negative regulator CARD8 (Ito et al., 2014). Small molecule inhibitors were shown to be beneficial in mice models of FCAS and MWS; the anti-inflammatory molecule BHB inhibits constitutive NLRP3 inflammasome activity (Youm et al., 2015). Additionally, the specific NLRP3 inhibitor MCC950 showed efficacy in mouse models of CAPS harboring activating NLRP3 mutations (Coll et al., 2015), and in mouse models for dermal and airway inflammation (Primiano et al., 2016).

**Familial Mediterranean fever** (FMF, OMIM #249100) is an autosomal recessive disorder caused by gain-of-function mutations in the *MEFV* gene encoding pyrin (French FMF Consortium, 1997). Pyrin contains a 14-3-3 binding motif which, when phosphorylated, regulates the compartmentalization (Jeru et al., 2005) and inhibits the activity of pyrin (Park et al., 2016). Pyrin mutations or inactivation of effector kinases by bacterial toxins leave the protein unphosphorylated and free to form a pyrin-inflammasome and activate caspase-1 (Chae et al., 2006; **Figure 1**). The current first-line treatment for FMF is colchicine, which, via RhoA effector kinases, can lead to pyrin phosphorylation and result in its inactivation (Park et al., 2016). Symptoms of FMF include periodic fever attacks, abdominal and chest pain, serositis, amyloidosis and cutaneous inflammation (Jesus and Goldbach-Mansky, 2014). Recently, a specific dominantly inherited S242R mutation in the 14-3-3 binding motif has been identified and shown to result in pyrin-associated autoinflammation with neutrophilic dermatosis (PAAND), an autoinflammatory disease with distinct clinical features such as severe recurrent neutrophilic dermatosis, fever and absence of serositis and amyloidosis (Masters et al., 2016).

In 2008, treatment of FMF using IL-1β antagonists was first reported in patient that received 50 mg/day Anakinra subcutaneously without interrupting colchicine. Fever attacks and chest pain were reduced during Anakinra treatment but reappeared upon discontinuation (Calligaris et al., 2008). Recent results of a double-blind, placebo-controlled, randomized study involving 14 colchicine-resistant FMF patients showed that those who received Anakinra daily at a subcutaneous dosage of 100 mg had significantly less fever attacks per month (1.7 vs. 3.5 in the placebo group) (ClinicalTrials.gov Identifier: NCT01705756) (Ben-Zvi et al., 2017).

Previously, Rilonacept was also shown to be a possible treatment option for colchicine-resistant or -intolerant FMF patients: in a small randomized, double-blind, alternating treatment study, Rilonacept given at 2.2 mg/kg weekly reduced the attack frequency to 0.77 per month in comparison to 2 per month in the placebo-treatment group. (ClinicalTrials.gov Identifier: NCT00582907) (Hashkes et al., 2012).

IL-1β inhibition with Canakinumab was also reported to be effective in colchicine-resistant FMF patients. In a 6-month, phase 2, open-label, single-arm study, seven children who experienced FMF attacks under daily colchicine treatment received three monthly subcutaneous injections of Canakinumab (2 mg/kg). The median attack rate per month decreased from 2.7 to 0.3 during the treatment period (ClinicalTrials.gov Identifier: NCT01148797) (Brik et al., 2014). In a second study, nine patients received three consecutive injections of 150 mg Canakinumab every 4 weeks. During the treatment period, only one patient had an attack (peritonitis) and five patients experienced an attack in the 2-months follow up period (ClinicalTrials.gov Identifier: NCT01088880) (Gül et al., 2015). In a retrospective longitudinal outcome study, the effects of long-term Canakinumab treatment in 14 colchicine-resistant FMF patients were assessed. All patients responded to the treatment but four relapsed during the follow-up. The shortening of Canakinumab administration intervals from 8/6 weeks to 4 weeks resulted in partial to full clinical remission (Laskari et al., 2017).

**Deficiency of IL-1 receptor antagonist** (DIRA, OMIM #612852) is very rare autoinflammatory disease with onset in the neonatal period and presents as systemic inflammation, pustular skin lesions, joint swelling, periostitis and multifocal osteomyelitis (Altiok et al., 2012). DIRA is caused by homozygous mutations in the *IL1RN* gene. It was first described in nine children harboring mutations leading to the synthesis of a truncated non-functional form of IL-1Ra (Aksentijevich et al., 2009; **Figure 1**). Around the same time, another group reported the case of a 49-day-old baby presenting a 175-kb homozygous deletion in chromosome 2 which was spaced over six IL-1 family members including *IL1RN*. This patient completely recovered after Anakinra treatment (Reddy et al., 2009). Another case report described the positive response to Anakinra in a 3 month-old child with confirmed DIRA (Schnellbacher et al., 2013).

More recently, treatment of a 12 year-old child suffering from DIRA due to a novel IL1RN mutation with 150 mg Canakinumab given every 6 weeks led to complete remission without side effects (Ulusoy et al., 2015).

A pilot open-label study to assess the efficacy of Rilonacept treatment for DIRA was completed in April 2016 but results are yet to be published (ClinicalTrials.gov Identifier: NCT01801449). Preliminary data on safety and efficacy in six patients suggests that a weekly injection of 4.4 mg/kg Rilonacept is required to achieve remission (Neal et al., 2014, meeting abstract).

**Tumor Necrosis Factor Receptor Associated Periodic Syndrome** (TRAPS, OMIM #142680) is an autosomal dominant inherited disorder linked to mutations of *TNFRSF1A* gene encoding the TNFα receptor 1 (McDermott et al., 1999; Hull et al., 2002). These mutations produce a misfolded receptor defective in shedding that accumulates in the cytoplasm and results in enhanced NF-κB activation, ROS production and impaired autophagy (Bachetti and Ceccherini, 2014). TRAPS symptoms include long-lasting (more than 1 week) fever associated with abdominal pain, skin lesions, and serositis. Various types of skin lesions occur, most frequently erythematous patches and plaques that can be migratory and associated with underlying myalgia.

Anti-TNF treatments were shown to be partially beneficial in TRAPS but may also cause paradoxical inflammatory attacks (Drewe et al., 2007). In contrast, IL-1 blockade seems to be more beneficial. Indeed, remarkable improvement was reported in TRAPS patients treated with Anakinra (Simon et al., 2004; Gattorno et al., 2008; Greco et al., 2015). Specifically targeting IL-1β was also shown to be successful for the treatment of TRAPS. In 2012, it was first reported that a woman who was taken off anti-TNF treatment and received 150 mg Canakinumab every 8 weeks instead, had complete remission (Brizi et al., 2012). Recently, the results of an open-label, proof-of-concept, phase 2 study were released: 20 patients received 150 mg Canakinumab every 4 weeks for 4 months. 19/20 patients achieved clinical remission at day 15 and all relapsed after withdrawal of the drug (ClinicalTrials.gov Identifier: NCT01242813) (Gattorno et al., 2017). Interestingly, a mutation or duplication of the *TNFRSF11A* gene coding for the receptor RANK was associated in three patients with recurrent episodes of fever. Analysis of serum from one patient revealed increased levels of inflammatory cytokines and particularly an eightfold increase for IL-18 (Jeru et al., 2014).

**Mevalonate kinase deficiency** (MKD) is an autosomal recessive metabolic disorder caused by mutations in the *MVK* gene (Haas and Hoffmann, 2006). Mevalonate kinase is an enzyme involved in the synthesis of cholesterol and isoprenoids. Mutations in this gene lead to shortage of geranylgeranylated proteins which cause the activation of the pyrin inflammasome and subsequent secretion of IL-1β (Mandey et al., 2006; van der Burgh et al., 2013; Park et al., 2016; **Figure 1**). Two forms of the disease exist. The less severe **hyperimmunoglobulinemia D syndrome** (HIDS; OMIM #260920) is characterized by sporadic fever episodes with skin lesions (widespread erythematous macules and papules), lymphadenopathy, abdominal and joint pain, diarrhea and headache (van der Meer et al., 1984). The rare, more severe form of the disease **mevalonic aciduria** (MVA; OMIM #610377) presents all above symptoms chronically (Berger et al., 1985).

In 2005, the case of a 38-year-old HIDS patient with recurrent fever episodes with symptom normalization following 100 mg/day Anakinra treatment was reported (Bodar et al., 2005). As fever episodes in HIDS occur at irregular intervals of 2–8 weeks (van der Burgh et al., 2013), Anakinra treatment “on-demand” appears to be an optimal mode of management with significant clinical response in 8 out 12 attacks (Bodar et al., 2011). Treatment of HIDS with Canakinumab was first reported in a 7-year-old child where 4 mg/kg administered every 4 weeks resulted in the prevention of fever attacks (Tsitsami et al., 2013). Recently, a retrospective study of 144 MKD patients described the response to different therapeutic approaches, including IL-1 antagonists. Anakinra given only during attacks resulted in three complete and five partial responses whereas out of the 19 patients who received Anakinra as maintenance therapy, 3 exhibited a complete remission, 13 a partial remission and 3 did not respond. Canakinumab led to complete remission in four patients and partial remission in a patient resistant to all other therapies (Ter Haar et al., 2016). In an open-label, single treatment arm study, 9 HIDS patients received Canakinumab every 6 weeks for 6 months followed by a withdrawal phase lasting up to 6 months and a 24 month-long-term treatment period. Canakinumab treatment reduced the frequency of flares from a median of 5 flares to 0 (unpublished results; Aróstegui et al., 2015, oral presentation) (ClinicalTrials.gov Identifier: NCT01303380).

**PAPA syndrome** (pyogenic arthritis, pyoderma gangrenosum, and acne, OMIM #604416) is a hereditary autosomal dominant autoinflammatory syndrome caused by gain-of-function mutations in the *PSTPIP1* gene (Lindor et al., 1997; Wise et al., 2002). The resulting mutated protein interacts with, and activates pyrin, causing dysregulated processing of IL-1β and IL-18 (Shoham et al., 2003; **Figure 1**). PAPA syndrome is characterized by early onset of recurrent sterile arthritis with neutrophilic infiltrates, with variable skin involvement including pyoderma gangrenosum and severe nodulo-cystic acne in adolescence and beyond.

In the first report of PAPA, Anakinra was administered to control arthritis flares in patients presenting the PAPA mutation while not presenting PG symptoms (Dierselhuis et al., 2005). Efficacy of Anakinra was subsequently reported again in a patient presenting the triad of symptoms; after 5 days of daily Anakinra administration at 100 mg per day, the skin lesions improved and after 1 month the ulcer due to PG completely healed with concomitant disappearance of arthritis and acne (Brenner et al., 2009).

The use of Canakinumab for the treatment of PAPA has also been reported: 150 mg Canakinumab given every 8 weeks led to complete healing of PG and disappearance of acne lesions in a single patient (Geusau et al., 2013). IL-18 levels were reported to be elevated in a PAPA syndrome patient treated with cyclosporine. Although PG was treated, acne and splenomegaly were not, suggesting a possible role for IL-18 (Kanameishi et al., 2016). The treatment of PAPA syndrome is challenging, however, since the response to therapy varies largely between patients.

**Blau syndrome** (BS, OMIM # 186580) is an autosomal dominant granulomatous disease caused by a mutation in *CARD15/NOD2* gene (Miceli-Richard et al., 2001). NOD2 is an intracellular receptor able to sense the bacterial peptidoglycan MDP (McDermott et al., 1999), and signals via the NF-κB pathway (Franchi et al., 2009). Mutations of *NOD2* are also linked to Crohn’s disease (CD; OMIM #266600) and early-onset sarcoidosis (EOS, OMIM #609464); in EOS and BS, mutations in the nucleotide-binding oligomerization domain results in increased NF-κB activity (Maekawa et al., 2016).

IL-1β inhibition was shown in individual case reports to be useful for the treatment of BS. Indeed, a patient treated with Anakinra exhibited inflammatory symptom improvement and normalization of plasma cytokine levels (Aróstegui et al., 2007). The use of Canakinumab was also reported for the treatment of Blau syndrome-related uveitis in a young patient and resulted in disease remission and stabilization of proinflammatory cytokine expression comparable to that seen in healthy controls (Simonini et al., 2013).

### Polygenic Autoinflammatory Diseases and Chronic Inflammatory Diseases

**PASH syndrome** (pyoderma gangrenosum, acne, and suppurative hidradenitis) is an autoinflammatory syndrome similar to but distinct from PAPA. It was first described in two patients presenting both pyoderma gangrenosum and acne without suffering from pyogenic arthritis (Braun-Falco et al., 2012). The genetic background of PASH syndrome is very heterogeneous; the absence of a *PSTPIP1* gene mutation was first reported, however, researchers recently found a *PSTPIP1* gene mutation in a PASH patient (Calderon-Castrat et al., 2016). Moreover, mutations in other genes involved in this autoinflammatory disease including *NLRP3, MEFV, NOD2*, and *NCSTN* have also been described in PASH (Marzano et al., 2014a; Duchatelet et al., 2015).

Treatment of PASH with Anakinra was reported in only one patient and resulted in partial remission (Braun-Falco et al., 2012).

In addition to PAPA and PASH, other phenotypically related syndromes are emerging. They include PASS (pyoderma gangrenosum, acne, suppurative hidradenitis, and axial spondyloarthritis), PAPASH (pyogenic arthritis, pyoderma gangrenosum, acne, and HS) and PsAPASH (psoriatic arthritis, pyoderma gangrenosum, acne, and HS) (Bruzzese, 2012; Marzano et al., 2013; Saraceno et al., 2015). Recently, a PASS patient treated with 100 mg/day Anakinra and improvement of all symptoms was reported, after Anakinra discontinuation relapse occurred within 3 days (Leuenberger et al., 2016).

**Schnitzler syndrome** (SchS) is a rare late-onset inflammatory disease considered as a sporadic acquired autoinflammatory disorder characterized by recurrent fever, urticarial skin lesions, arthritis and lymphadenopathy accompanied with IgM gammopathy. Treatment with Anakinra was reported to completely abrogate the symptoms within 24 h in several case reports (de Koning et al., 2006; Dybowski et al., 2008; Sonnichsen et al., 2016).

In a prospective, open-label study, all patients receiving Rilonacept for up to 1 year showed a rapid clinical response over the treatment duration with nearly complete remission in four of eight patients (ClinicalTrials.gov Identifier: NCT01045772) (Krause et al., 2012).

Canakinumab treatment in SchS was first positively reported in a patient switching from Anakinra (de Koning et al., 2011). In an open-label, single-treatment arm trial, eight additional patients switched from Anakinra to monthly injections of 150 mg Canakinumab for 6 months. Clinical remission was observed in all patients at day 14 and lasted up to 6 months (full duration of the trial) in seven out of eight patients (ClinicalTrials.gov NCT01276522) (de Koning et al., 2013). In another case, injection of Canakinumab every 8 weeks resulted in the disappearance of the symptoms until drug withdrawal (ClinicalTrials.gov Identifier: NCT01245127) (Vanderschueren and Knockaert, 2013). Recently, a phase 2, randomized placebo-controlled, multi-center trial including 20 patients confirmed the potential of Canakinumab for the treatment of SchS. 7 days after initial injection, 5/7 patients who received the drug showed significant improvement when compared to placebo treated patients (0/13). In the open-label trial phase, all patients received Canakinumab, and after 14 days, 15/20 exhibited complete remission and 5 partial remission (ClinicalTrials.gov Identifier: NCT01390350) (Krause et al., 2017).

**Hidradenitis suppurativa** (HS; OMIM #142690, #613736, #613737), also known as acne inversa, is a chronic skin disease of the hair follicles affecting the axillary, inguinal and anogenital regions with formation of nodules and abscesses (Kurzen et al., 2008). Mutations in the γ-secretase genes *NCSTN, PSENEN*, and *PSEN1* impairing the Notch signaling in hair follicles have been found in some HS patients (Pink et al., 2012). TNF-α, IL-1β, and IL-10 levels were frequently increased in HS lesions (van der Zee et al., 2011). Moreover, IL-17, caspase-1 and NLRP3 are elevated in lesions of HS skin (Lima et al., 2016).

The use and success of Anakinra for the treatment of HS has been a matter of controversy (van der Zee and Prens, 2013; Zarchi et al., 2013; Leslie et al., 2014; Menis et al., 2015; Russo and Alikhan, 2016). However, in a recent double-blind, randomized, placebo controlled trial, 20 patients were treated with Anakinra or placebo daily for 12 weeks. HS clinical response after 12 weeks was 78% in Anakinra-treated patients versus 30% in the placebo group (ClinicalTrials.gov Identifier: NCT01558375) (Tzanetakou et al., 2016).

Treatment with Canakinumab was reported in one HS patient with concomitant PG. HS healed after the first injection and PG after 4 months of treatment (Jaeger et al., 2013). Recently a double-blind, randomized, placebo-controlled clinical trial investigating the efficacy of anti-IL-1α therapy in HS patients who were refractory to anti-TNF drugs was completed. Patients receiving MaBp1 every 2 weeks for 12 weeks showed a 60% response rate in comparison to 10% in the placebo group (ClinicalTrials.gov Identifier: NCT02643654) (Xbiotech, 2017).

**SAPHO syndrome** (synovitis, acne, pustulosis, hyperostosis, osteitis) is a chronic inflammatory disorder targeting bones, skin and joints (Chamot et al., 1987). Skin manifestations include palmoplantar pustulosis, psoriasis, severe acne and HS (Firinu et al., 2016).

Dysregulation of the ATP receptor P2X_7_ in SAPHO PBMCs causes in increased processing of IL-1β suggesting a possible therapeutic approach: 100 mg/day Anakinra treatment in a 47-year-old female resulted in the disappearance of the symptoms within 3 months (Colina et al., 2010). In a short-term open study 6 SAPHO patients received 100 mg/day Anakinra, with clinical response reported in 5/6 patients (Wendling et al., 2012).

**Behçet’s disease** (BD) is a chronic multisystem disease that features vasculitis leading to clinical symptoms comprising bipolar aphtosis (oral and genital), uveitis, polyarthritis and skin lesions including sterile non-follicular pustules on the skin and erythema nodosum (Mazzoccoli et al., 2016). The etiology of BD is still unclear but there is an association with genetic factors like human leukocyte antigen (HLA)-B51 or the extrinsic factor heat shock protein from *Streptococcus sanguinis* which could activate the innate immune system via TLR signaling (Alpsoy, 2016). Anti-IL-1β therapy is an effective treatment of Behçet’s disease. Treatment with Anakinra of nine BD patients refractory to anti-TNF resulted in a response in eight individuals (Cantarini et al., 2015). Treatment of three BD patients with 150 mg Canakinumab every 6–8 weeks resulted in complete remission of all clinical manifestations and relapse was not observed at long-term follow up (Vitale et al., 2014). A recent retrospective study showed that the treatment of patients with Canakinumab or Anakinra for at least 12 months led to complete and sustainable remission of the disease (Emmi et al., 2016) and was also effective for BD-related uveitis (Fabiani et al., 2016).

A phase 3 clinical trial evaluating Gevokizumab for the treatment of patients with BD uveitis was terminated because it did not meet the primary endpoint criteria. However, decreased disease severity was observed in the setting of this trial (ClinicalTrial.gov Identifier: NCT01965145) (Xoma, 2015).

### Neutrophilic Dermatoses

**Pyoderma gangrenosum** (PG) is a rare non-infectious neutrophilic dermatosis characterized by sterile pustular skin lesions that rapidly evolve into tender skin ulcers with undermined borders of varying size and depth, sometimes exposing underlying tendons or muscles. PG is frequently associated with systemic diseases. Increased expression of IL-1β was found in lesional skin of PG patients compared to healthy skin (Marzano et al., 2014b; Kolios et al., 2015). In an open-label, proof of concept study evaluating the anti-IL-1β monoclonal antibody Gevokizumab, six patients with active ulcers received three subcutaneous injections once every 4 weeks and four out of six patients had a complete clearance of the target ulcer, 1 a partial (90%) closure of the ulcer and 1 did not respond (ClinicalTrials.gov Identifier: NCT01882504) (Huang et al., 2014, poster). A phase 3 trial of the same drug was prematurely terminated after the company’s decision to interrupt clinical development of Gevokizumab. However, preliminary results with 25 patients treated with gevokizumab did not apparently reveal any significant benefit (ClinicalTrials.gov Identifier: NCT02326740 and NCT02315417) (Xoma, 2016). Canakinumab treatment was first reported in a PG patient with concomitant HS. Ulceration disappeared after 4 months and complete remission was achieved after 12 months of treatment (Jaeger et al., 2013). In an open-label study, five steroid-refractory PG patients were treated with Canakinumab once with 150 mg at onset and then optionally at weeks 2 and 8 if response was suboptimal. At the week 16 study endpoint 80% of the patients showed decreased size of target ulcers and 60% were in complete remission (ClinicalTrials.gov Identifier: NCT01302795) (Kolios et al., 2015). A case report described complete healing of a PG patient that received a Canakinumab monthly at a 150 mg dose for 3 months (Galimberti et al., 2016). The involvement of IL-1α in the pathogenesis of PG is currently being investigated in a phase 2 open label study of MABp1 (ClinicalTrials.gov Identifier: NCT01965613) (unpublished data).

**Sweet’s syndrome** (SwS) or acute febrile neutrophilic dermatosis, is a neutrophilic dermatosis with systemic symptoms characterized by fever, tender red cutaneous nodules or papules, occasionally covered with vesicles, pustules or bullae, usually affecting the upper limbs, face and neck. SwS is frequently observed in patients with leukemia or connective tissue diseases. Overexpression of proinflammatory genes including IL-1β is reported in lesional skin of SwS patients (Marzano et al., 2014b; Imhof et al., 2015).

Anakinra (100 mg/day) resulted in symptom resolution within 4 days and subsequent remission for 19 months in one case reported (Delluc et al., 2008). Another case report described disappearance of skin lesions within a month of Anakinra treatment and reappearance of symptoms upon withdrawal (Kluger et al., 2011).

**Amicrobial pustulosis of the skin folds** (APF) is a rare, chronic cutaneous disease presenting aseptic pustular lesions in cutaneous folds and usually occurring in young women affected by autoimmune diseases such as SLE (Marzano et al., 1996).

Treatment of APF with Anakinra was described in one patient who had increased levels of IL-1α expression in lesional skin, was refractory to steroid therapy and TNF antagonists. Daily subcutaneous injection of Anakinra for 1 month resulted in clearance of the lesions (Amazan et al., 2014).

### Other Diseases with Skin Involvement

#### Acne Vulgaris

Acne vulgaris is a common inflammatory and potentially severe skin disease associated with colonization of the pilo-sebaceous unit by the commensal bacterium *P. acnes*. *P. acnes* is considered to contribute to inflammation in acne and has been shown to activate the NLRP3 inflammasome in human monocytes (Kistowska et al., 2014b; Qin et al., 2014) and in sebocytes (Li et al., 2014). Therefore, IL-1β is thought to play an important role in acne pathogenesis. Gevokizumab was evaluated in a double-blind, randomized, placebo-controlled phase 2 trial for the treatment of inflammatory facial lesions. Patients who received 0.6 mg/kg Gevokizumab once a month for 3 months showed a significant clinical response associated with reduction of inflammatory acne lesions in comparison to the control group (ClinicalTrial.gov Identifier: NCT01498874) (Xoma, 2013). On the other hand, an open label, phase 2 study testing the anti-IL-1α antibody MABp1 on 11 patients showed a 36% decrease in lesion counts (Carrasco et al., 2015).

#### *Malassezia-*Associated Skin Diseases

The fungal genus *Malassezia* is linked to several inflammatory skin diseases such as seborrheic dermatitis (seborrheic eczema), pityriasis versicolor (tinea versicolor), atopic eczema, psoriasis, *Malassezia* folliculitis and Onychomycoses (Gaitanis et al., 2012). The etiological agent of pityriasis versicolor, *Malassezia* was shown to activate the NLRP3 inflammasome via the dectin-1 and Syk signaling cascade, causing the release of IL-1β (Kistowska et al., 2014a). To date, no IL-1 blocker has been evaluated for the treatment of *Malassezia-*associated skin diseases.

**Psoriasis** is an immune-mediated inflammatory disease that affects 2–3% of the global population. It affects primarily the skin and the joints. Psoriasis vulgaris manifests as red, scaly patches of the skin. In lesions, keratinocytes express IL-1α, IL-1β, and IL-18 which regulate the expression of genes involved in the pathogenesis of psoriasis including S100A7 and LL-37 (Perera et al., 2012). By binding to cytosolic DNA, LL-37 has been shown to impair the activation of the AIM2 inflammasome, which is highly expressed in psoriatic lesions (Dombrowski et al., 2011). However, LL-37 is able to induce secretion of IL-18 from keratinocytes independently of caspase-1 (Niyonsaba et al., 2005). IL-1α inhibition for the treatment of plaque psoriasis was investigated in a small size (eight patients), open-label, single-arm trial. 200 mg of MABp1 injected subcutaneously every 3 weeks until evaluation at day 56 showed an average of 13% decrease of the PASI score (Coleman et al., 2015), which is lower than that obtained with the current approved therapies using anti-TNF, -IL-17A, and -IL-12/IL-23 (Mansouri and Menter, 2015).

Generalized pustular psoriasis (GPP; OMIM # 614204, # 602723) is a rare, severe form of psoriasis caused by mutations in the *IL36RN* and *CARD14* genes. Treatment of GPP with Anakinra (Viguier et al., 2010) and Gevokizumab (Mansouri et al., 2015) resulted in a reduction in GPP area and severity index.

Moreover, the caspase-1 inhibitor Belnacasan (VX-765) was evaluated in a phase 2a trial against psoriasis but patients did not respond to this therapy (ClinicalTrial.gov Identifier: NCT00205465) (Vertex, 2011).

**Systemic juvenile idiopathic arthritis (sJIA)** is a juvenile form of polyarticular arthritis that also presents with systemic symptoms including fever and skin lesions. Standard treatment includes non-steroidal anti-inflammatory drugs, corticosteroids, anti-IL-1 and anti-IL-6 (Cimaz, 2016).

IL-1 blockade with Anakinra, Canakinumab, and Rilonacept has shown positive results in three clinical trials by Quartier et al. (2011) (ClinicalTrials.gov Identifier: NCT00339157), and Ruperto et al. (2012) (ClinicalTrials.gov Identifier: NCT00886769) and Lovell et al. (2013) (ClinicalTrials.gov Identifier: NCT01803321), respectively. These studies are extensively discussed in the review by Giancane et al. (2016) in this issue.

**Adult-onset Still’s disease (AoSD)** is a rare form of inflammatory arthritis that shares symptoms with sJIA but presents during adulthood.

Treatment of AoSD with Anakinra has shown efficacy in several studies (Castaneda et al., 2016). In an open, randomized study involving 22 patients, Anakinra treatment was compared to disease-modifying anti-rheumatic drugs (DMARDs). Patients receiving ≥10 mg/day of Anakinra showed a better overall response in comparison to DMARDs (ClinicalTrials.gov Identifier: NCT01033656) (Nordstrom et al., 2012). In another report, three patients with refractory AoSD that were switched from Anakinra to Rilonacept showed prolonged complete remission (Petryna et al., 2012). Treatment of AoSD with Canakinumab was first reported in two patients resistant to Anakinra and resulted in improvement of both systemic symptoms and polyarthritis (Kontzias and Efthimiou, 2012). In another report, a patient receiving 150 mg Canakinumab every 8 weeks showed long-term improvement of systemic symptoms but active arthritis persisted up to 14 months follow-up (Lo Gullo et al., 2014).

**Graft-versus-host disease** (GvHD) is a severe complication after allogeneic hematopoietic stem cell transplantation (allo-HSCT). Acute GvHD occurs in 35–50% of transplanted patients and about half of them will eventually develop chronic GvHD (Jacobsohn and Vogelsang, 2007). Skin manifestations include erythema, morbilliform exanthema, and confluent erythroderma, but in the severest forms (Grade IV) widespread skin detachment (Lipsker et al., 2016). First-line treatment for GvHD consists of corticosteroids and calcineurin inhibitors followed by anti-TNF, anti-IL-2 or mTOR inhibitors (Dignan et al., 2012).

The efficacy of Anakinra in the treatment of GvHD was assessed in 1994 in an open-label, phase 1/2 trial of 17 steroid-resistant GvHD patients. Anakinra was continuously administered per infusion for 1 week. In 63% of the patients, acute GvHD improved by at least one grade (Antin et al., 1994). A second double-blind, placebo-controlled randomized trial on 181 patients investigated the role of IL-1 in the initial T-cell mediated development of the disease by giving Anakinra during conditioning (4 days) and for 10 days after allo-HCT. There was no difference between IL-1ra- and placebo-treated patients with 61 and 59% of them, respectively, developing moderate to severe GvHD (Antin et al., 2002).

Recently, in a murine model of acute GvHD, it was demonstrated that conditioning therapy before allo-HCT resulted in NLRP3 activation in the recipient. Microflora translocation and uric acid released by dying cells were able to activate the inflammasome. Inhibition of NLRP3 with glibenclamide, the IL-1β antagonist Anakinra or gene deletion of *Nlrp3* or *Asc* in mice resulted in delayed and reduced mortality (Jankovic et al., 2013).

Similarly, the blockade of IL-18R in mice has been shown to prevent the early phase of GvHD pathogenesis (Li et al., 2015).

## Conclusion

Thanks to the discovery of the inflammasome and to major advances in the understanding of biological properties and clinical relevance of IL-1 family members,’ the use of IL-1 antagonists has been quite intensely investigated for the treatment of inflammatory and autoinflammatory diseases (**Table 2**). The introduction of IL-1 antagonists represents a major breakthrough in the management of several autoinflammatory diseases, including not only cryopyrinopathies but also other inflammatory conditions refractory to standard therapies where neutrophils play an important pathogenic role. Clinical responses to IL-1β antagonists suggest that this cytokine plays a critical role in the pathogenesis of autoinflammatory disorders. Indeed, many studies have demonstrated that there is no loss in therapeutic efficacy when Anakinra is substituted with the IL-1β-specific antagonist Canakinumab, suggesting that in comparison to IL-1α and/or IL-18, IL-1β likely plays a predominant role in a substantial number of diseases described in this review.

**Table 2:** Selected clinical trials targeting IL-1 in inflammatory skin diseases.

Syndrome	Drug	CTI	*n*	Ph	Design	Reference
CINCA	Anakinra	NCT00069329	18	1	O, W	Goldbach-Mansky et al., 2006
FCAS	Anakinra	NCT00214851	8	1	O	Ross et al., 2008
FCAS	Rilonacept	NCT00094900	5	2	O	Goldbach-Mansky et al., 2008
FCAS/MWS	Rilonacept	NCT00288704	47	3	R-B-P, W	Hoffman et al., 2008
CAPS	Canakinumab	NCT00465985	35	3	O, R-B-P, W	Lachmann et al., 2009
MWS/CINCA	Canakinumab	NCT00487708	34	2	O	Kuemmerle-Deschner et al., 2011b
CAPS	Canakinumab	NCT00685373	166	3	O	Kuemmerle-Deschner et al., 2011a
CINCA	Canakinumab	NCT00770601	6	3	O	Sibley et al., 2015
CAPS	Canakinumab	NCT00991146	19	3	O	Yokota et al., 2016
**CAPS**	**Canakinumab**	**NCT01213641**	**288**	**pr**	**O**	Hoffman et al., 2016
FMF	Anakinra	NCT01705756	25	3	R-B-P	Ben-Zvi et al., 2017
FMF	Rilonacept	NCT00582907	14	2	R-B-AT	Hashkes et al., 2012
FMF	Canakinumab	NCT01148797	7	2	O	Brik et al., 2014
FMF	Canakinumab	NCT01088880	9	2	O	Gül et al., 2015
**DIRA**	**Rilonacept**	**NCT01801449**	**6**	**2**	**O**	Neal et al., 2014
TRAPS	Canakinumab	NCT01242813	20	2	O, W	Gattorno et al., 2017
**MKD/HIDS**	**Canakinumab**	**NCT01303380**	**9**	**2**	**O, W**	Aróstegui et al., 2015
SchS	Rilonacept	NCT01045772	8	2	O	Krause et al., 2012
SchS	Canakinumab	NCT01276522	8	2	O	de Koning et al., 2013
SchS	Canakinumab	NCT01245127	1	2	O	Vanderschueren and Knockaert, 2013
SchS	Canakinumab	NCT01390350	20	2	R-B-P, O	Krause et al., 2017
HS	Anakinra	NCT01558375	20	2	R-B-P	Tzanetakou et al., 2016
**HS**	**MABp1**	**NCT02643654**	**20**	**2**	**R-B-P**	Xbiotech, 2017
BD (uveitis)	Gevokizumab	NCT01965145^†^	83	3	R-B-P	Xoma, 2015
**PG**	**Gevokizumab**	**NCT01882504**	**6**	**2**	**O**	Huang et al., 2014
PG	Gevokizumab	NCT02326740^†^	9	3	R-B-P, O	Xoma, 2016
PG	Gevokizumab	NCT02315417^†^	16	3	R-B-P, O	Xoma, 2016
PG	Canakinumab	NCT01302795	5	2	O	Kolios et al., 2015
**PG**	**MABp1**	**NCT01965613**	**10**	**2**	**O**	na
Acne vulgaris	Gevokizumab	NCT01498874	127	2	R-B-P	Xoma, 2013
Acne vulgaris	MABp1	na	11	2	O	Carrasco et al., 2015
Psoriasis	Belnacasan	NCT00205465	64	2	R-B-P	Vertex, 2011
sJIA	Anakinra	NCT00339157	24	2	R-B-P	Quartier et al., 2011
sJIA	Rilonacept	NCT01803321	24	1	R-B-P	Lovell et al., 2013
sJIA	Canakinumab	NCT00886769	84	3	R-B-P	Ruperto et al., 2012
AoSD	Anakinra	NCT01033656	22	2	R-O-CD	Nordstrom et al., 2012

*CTI, ClinicalTrial.gov Identifier; n, number of enrolled individuals; Ph, phase; pr, prospective; R, randomized; O, open-label; B, double-blind; P, placebo-controlled; W, withdrawal phase; AT, alternating treatment; CD, comparator drug; na, not available. In bold: studies having only preliminary or not peer-reviewed results. ^†^Clinical trial was terminated.*

Due to the rarity of autoinflammatory syndromes, sample size represents a major limitation of clinical studies. Nevertheless, retrospective studies, including online registers such as the β*-confident register* for CAPS, that collect data from several reports have definitively helped establishing solid data on the efficacy and safety of IL-1 antagonists for these pathologies.

## Author Contributions

GF collected and reviewed the literature, drew the figure and wrote the manuscript. EC and LF gave valuable and professional suggestions and revised the manuscript. All authors approved the final version of the manuscript.

## Conflict of Interest Statement

The authors declare that the research was conducted in the absence of any commercial or financial relationships that could be construed as a potential conflict of interest.
